# Glomerular Biomechanical Stress and Lipid Mediators during Cellular Changes Leading to Chronic Kidney Disease

**DOI:** 10.3390/biomedicines10020407

**Published:** 2022-02-09

**Authors:** Mukut Sharma, Vikas Singh, Ram Sharma, Arnav Koul, Ellen T. McCarthy, Virginia J. Savin, Trupti Joshi, Tarak Srivastava

**Affiliations:** 1 Research and Development Service, Kansas City VA Medical Center, Kansas City, MO 64128, USA; Ram.sharma2@va.gov; 2 Midwest Veterans’ Biomedical Research Foundation, Kansas City, MO 64128, USA; akoul2018@gmail.com (A.K.); vjsavin@gmail.com (V.J.S.); tsrivastava@cmh.edu (T.S.); 3 Department of Internal Medicine, The Jared Grantham Kidney Institute, University of Kansas Medical Center, Kansas City, MO 66160, USA; emccarthy@kumc.edu; 4 Neurology, Kansas City VA Medical Center, Kansas City, MO 64128, USA; Vikas.Singh3@va.gov; 5 Department of Health Management and Informatics, University of Missouri, Columbia, MO 65201, USA; Joshitr@missouri.edu; 6 Section of Nephrology, Children’s Mercy Hospital and University of Missouri, Kansas City, MO 64108, USA; 7 Department of Oral and Craniofacial Sciences, School of Dentistry, University of Missouri, Kansas City, MO 64108, USA

**Keywords:** hyperfiltration, biomechanical forces, podocytes, tubules, omega-6, omega-3, polyunsaturated fatty acids, lipid signaling, eicosanoids

## Abstract

Hyperfiltration is an important underlying cause of glomerular dysfunction associated with several systemic and intrinsic glomerular conditions leading to chronic kidney disease (CKD). These include obesity, diabetes, hypertension, focal segmental glomerulosclerosis (FSGS), congenital abnormalities and reduced renal mass (low nephron number). Hyperfiltration-associated biomechanical forces directly impact the cell membrane, generating tensile and fluid flow shear stresses in multiple segments of the nephron. Ongoing research suggests these biomechanical forces as the initial mediators of hyperfiltration-induced deterioration of podocyte structure and function leading to their detachment and irreplaceable loss from the glomerular filtration barrier. Membrane lipid-derived polyunsaturated fatty acids (PUFA) and their metabolites are potent transducers of biomechanical stress from the cell surface to intracellular compartments. Omega-6 and ω-3 long-chain PUFA from membrane phospholipids generate many versatile and autacoid oxylipins that modulate pro-inflammatory as well as anti-inflammatory autocrine and paracrine signaling. We advance the idea that lipid signaling molecules, related enzymes, metabolites and receptors are not just mediators of cellular stress but also potential targets for developing novel interventions. With the growing emphasis on lifestyle changes for wellness, dietary fatty acids are potential adjunct-therapeutics to minimize/treat hyperfiltration-induced progressive glomerular damage and CKD.

## 1. Introduction

### 1.1. Glomerular Hyperfiltration Is an Early Response That May Turn Maladaptive

Plasma filtration in the glomerulus and ultrafiltrate processing in the tubular segment are primary physiological functions of the nephron. Capillary endothelial cells, mesangial cells, glomerular basement membrane (GBM) and podocytes interact to constitute the glomerular filtration barrier modulated by multiple hemodynamic, neurohumoral and immune factors. Consistent glomerular filtration is essential for maintaining homeostasis and estimated glomerular filtration rate (eGFR) is an important clinical parameter for evaluating kidney function. Estimated GFR between 120–90 mL/min/1.73 m^2^ reflecting a physiological decline with age is considered normal for healthy adults (20–70 years). However, eGFR below 60 mL/min/1.73 m^2^ indicates chronic kidney disease (CKD) with moderate (59–30 mL, Stage 3) to severe (29–15 mL, Stage 4) loss of kidney function and kidney failure (Stage 5 CKD) below 15 mL/min/1.73 m^2^ [1,2].

Higher eGFR reflects an increased rate of glomerular filtration, termed glomerular hyperfiltration (GHF). A precise definition, threshold levels/range and an exact mechanism of GHF are subjects of ongoing studies and discussion. GHF is variously defined based on high whole kidney GFR, elevated filtration fraction (GFR/Renal blood flow *100) or increased single nephron glomerular filtration rate (SNGFR) to describe total renal filtration capacity. In large population studies, eGFR above the 95th or 97th percentile of the cohort is also used as the threshold value for GHF. A median threshold value of 135 mL/min/1.73 m^2^ (range 90.7–175 mL, most values between 135 and 140 mL) has been reported [3]. A recent study on diabetes defined GHF at GFR ≥140 mL/min per 1.73 m^2^, with secondary thresholds of 130 or 150 mL/min per 1.73 m^2^ [4]. Others have used 120 mL/min/1.73 m^2^ as the cutoff for GHF [5] which is closer to the upper limit of eGFR in healthy adults (90–120 mL).

**Figure 1:**
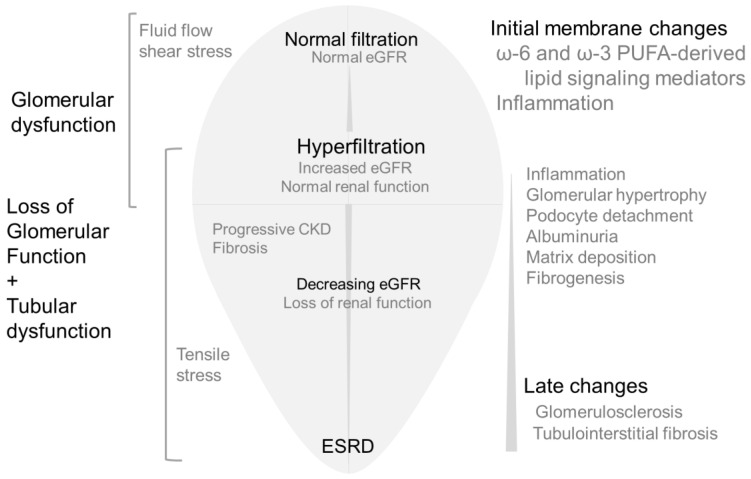
**Simplified version of renal changes caused by hyperfiltration followed by lower filtration.**Middle: Initially, glomerular filtration rate increases (hyperfiltration), followed by a decrease in GFR and CKD leading to ESRD. Left: Increased fluid flow shear stress drives effects of hyperfiltration in the early stages indicated by glomerular/podocyte dysfunction. Gradual increase in tensile stress is associated with rapid loss of glomerular function and tubular changes causing CKD. Right: Cellular stress causes inflammatory changes and the release of fatty acids from membrane phospholipids. Fatty acid metabolites mediate mechanotransduction and activate cellular signaling pathways as an initial response to hyperfiltration. With time, tubular homeostasis also changes in response to early glomerular changes. Initial lipid-mediated signaling events are followed by more complex and diverse signaling and functional changes resulting in albuminuria, matrix accumulation, fibrogenesis, podocyte loss leading to glomerulosclerosis and fibrosis.

An adaptive increase in GFR during pregnancy occurs due to physiological endocrine changes that result in higher cardiac output and lower peripheral resistance causing increased renal blood flow and glomerular size [6,7]. A transient physiological increase in GFR also occurs after consuming high-protein meals. Long-term high protein consumption may cause adverse renal effects in vulnerable groups such as obese individuals with subclinical renal damage [8,9,10]. Initial adaptive increase in GFR causes transient hemodynamic changes. However, adaptive changes may turn maladaptive and pathological due to persistent insult. Glomerular hyperfiltration followed by a gradual decline in filtration parallel structural changes in the glomerular and tubular compartment are outlined in Figure 1.

### 1.2. Glomerular Hyperfiltration Is Associated with Several Pathophysiological Etiologies

Maladaptive GHF, while not unique to a specific condition, is an underlying risk factor of early glomerular dysfunction in several diseases where normal kidneys are vulnerable to systemic or primary glomerular pathophysiological changes. GHF has been mostly described in the context of the early stages of obesity, diabetes and hypertension that are components of the metabolic syndrome spectrum which represents a co-occurrence of metabolic risk factors (abdominal obesity, hyperglycemia, dyslipidemia and hypertension) for type 2 diabetes and cardiovascular disease (CVD) [11,12]. GHF in humans may also be associated with hyperperfusion independent of blood pressure or diabetes [13]. GHF has been described in several other diseases that range from non-alcoholic fatty liver disease (NAFLD) to dementia and stroke. A lower nephron number due to unilateral renal agenesis or reduced functional renal mass due to other congenital abnormalities cause hyperfiltration early in life. Kidney donation also results in hyperfiltration leading to CKD after 20+ years in 3–10% of donors [14,15,16,17]. The growing number of studies involving subjects with single functional kidneys and relevant animal models of hyperfiltration will add valuable information to diabetes, obesity, and hypertension studies. Table 1 provides a summary of the conditions associated with GHF. Each of these conditions, with its unique features and variations, is a subject of detailed studies, some of which are cited.

### 1.3. Glomerular Hyperfiltration Is a Potential Predictor of CKD and Cardiovascular Disease

In addition to decreased eGFR, elevated blood pressure and microalbuminuria are clinically used indicators of kidney damage and decreased function. Albuminuria is the benchmark indicator of glomerular dysfunction, and it directly exacerbates tubular dysfunction due to oxidative stress, thus adding to the deterioration of renal function. Proteinuria at >1 g/day is prognostic of progressive renal disease, cardiovascular disease (CVD), and poor outcome. Nevertheless, these indicators represent existing low renal function and at least minimum structural change.

Increased glomerular filtration is an adaptive response mounted during the early stages, prior to functional loss. GHF is considered a surrogate marker of elevated intraglomerular pressure in patients with diabetes mellitus [94] and predictor of CKD [27] and it associates with increased risk of cardiovascular disease and all-cause mortality [95,96,97]. However, a lack of detailed understanding of early hyperfiltration and its mechanism has perhaps hindered the use of hyperfiltration as a clinical indicator of renal dysfunction.

### 1.4. Glomerular Hyperfiltration Precedes Tissue Fibrosis and Organ Failure

Hyperfiltration and renal fibrosis are temporally distinct events in the development and progression of CKD. Cellular stress caused by hyperfiltration induces changes in the plasma membrane, leading to signaling for mechanotransduction. These early events initiate increasingly complex responses that lead to fibrogenesis and end-stage renal disease (ESRD). Early events also trigger the synthesis of matrix proteins that begin to accumulate in the absence of matched degradation by metalloproteases. Continued matrix accumulation triggers uncontrolled and irreversible pathophysiological changes [98,99,100,101]. 

Initial signaling events associated with GHF are mediated mainly by membrane lipids, while subsequent fibrogenesis stimulates and links multiple signaling molecules. Inflammation and TGF-β/Smad signaling play a central role in fibrogenesis. Other key molecules and pathways related to fibrogenesis include fibroblast growth factor (FGF), platelet-derived growth factor, IL-1, TNF-α, renin-angiotensin- aldosterone system (RAAS), microRNA clusters, vitamin D, bradykinin, parathyroid hormone, eNOS and JAK/STAT pathway. Altered expression of membrane glycosaminoglycans (heparan sulfate, hyaluronic acid, etc.) also add to fibrogenesis by influencing the interactions between various effector molecules and cell receptors [102,103,104]. Thus, early intervention during hyperfiltration may slow down/prevent fibrogenesis.

### 1.5. Outline of the Article

This article focuses on GHF as an early event in renal disease associated with a variety of etiologies. A brief description of hyperfiltration in terms of the forces that cause cellular stress and affect glomerular function, as well as the significance of glomerular-tubular interaction, is provided. The description of the effect of GHF will focus on the initial response to physical forces at the plasma membrane. Membrane lipid-derived polyunsaturated fatty acids (PUFA) generate active mediators that drive the early cellular response by upregulating pro-inflammatory signaling. PUFA also serve as precursors of the specialized pro-resolving mediators (SPM) that resolve inflammation. Figures provide generalized summaries of metabolite synthesis, receptors and signaling. A brief section summarizes the ongoing efforts to target GHF using pharmacotherapeutics or by lifestyle and dietary changes. While the reported early changes in glomerular function and podocytes are central to this discussion, relevant information on other tissues/cells is also included.

## 2. Hyperfiltration and Biomechanical Forces

Figure 1 summarizes the temporal changes in renal structure and function caused by the hyperfiltration-induced increase in biomechanical forces. Hyperfiltration results from changes in the physicochemical characteristics of the filtration barrier due to increased afferent arteriolar dilation or efferent arteriolar constriction. These changes in the capillary are generally modeled assuming the capillary as a thin curved cylinder. This model provides a calculated difference in hydrostatic pressure at 40 mm Hg between glomerular capillary pressure (~55 mm Hg) and Bowman space (~15 mm Hg). Elevated capillary pressure increases biomechanical forces generating (i) axial and circumferential stress in the capillary walls that is balanced by the podocyte foot processes covering the capillary surface and by (ii) increased flow of the ultrafiltrate which, in turn, generates fluid flow shear stress (FFSS) on podocytes and their processes [105,106].

### 2.1. Fluid Flow Shear Stress (FFSS)

Fluid flow along the cell body and processes of podocytes is visualized as blood flow in the capillary and modeled using the flow of fluid over the flat surface of an object such as between two parallel plates or as fluid flow in a cylinder. Approximately 180 L/day of ultrafiltrate flows through human glomerular Bowman’s space that generates shear stress, causing cellular deformation of podocytes [105,106,107,108]. Fundamental considerations that form the basis of generating the plasma ultrafiltrate through the glomerular filtration barrier are based on hemodynamic parameters studied and established during the past several decades.

#### 2.1.1. The Glomerular Filtration Barrier Function

Briefly, renal blood flow, ultrafiltration pressure (P_UF_), and ultrafiltration coefficient (K_f_) are the main determinants of glomerular filtration. K_f_ (product of area and hydraulic permeability [L_p_]) determines the Single Nephron Glomerular Filtration Rate (SNGFR) as shown in the equation.

$$\mathrm{SNGFR}=K_{f}\int_{0{}^{\circ}}^{1{}^{\circ}}[(P_{g\left(x\right)}-P_{B})\;-\;\sigma \;(\pi_{g\;\left(x\right)}\;-\;\pi_{B})]\;dx$$

In the above equation, x represents the normalized length of the glomerular capillary with the afferent-end designated by 0 and the efferent-end by 1; P and π represent the hydraulic and osmotic pressures in glomerular capillary (g) and Bowman space (B), respectively, at distance x along the capillary length; σ symbolizes the reflection coefficient (range 0 to 1). This equation shows that increase in $K_{f}$ (determined by L_p_ and area) and/or ∆P (determined by P_GC_ and ∆π) mainly determine the increase in single-nephron glomerular filtration rate (SNGFR) associated with glomerular hyperfiltration.

#### 2.1.2. Unilateral Nephrectomy in Rodent Models of Hyperfiltration Increases Single-Nephron Glomerular Filtration Rate (SNGFR)

Hemodynamic parameters are utilized to study kidney disease in human subjects and animal models. Unilaterally nephrectomized (UNX) rodent models are valuable in determining hemodynamic changes and validating the calculated SNGFR in humans. Early studies showed that UNX in 5-day old rats (Sprague–Dawley) resulted in elevated SNGFR and P_UF_ by 20 days of age, increased glomerular area and decreased Lp by 60 days of age. These findings also demonstrated that high ultrafiltration pressure was not an absolute requirement for increasing SNGFR in UNX rats. Additional studies showed that SNGFR increased by 30–36% in mice and 57–86% in rats [109,110,111]. Recent studies in our laboratory using mathematical modeling showed a 2-fold increase in SNGFR, a 2-fold increase in FFSS in UNX rats and a 2- to 3-fold increase in FFSS in UNX mice [73].

#### 2.1.3. FFSS Mediates the Early Effects of Hyperfiltration

Shear stress due to ultrafiltrate flow targets the cell body and primary processes [107,112] and FFSS is considered the main cause of podocyte detachment and irreplaceable loss [112,113]. In this regard, FFSS-treated cultured podocytes show arrangement of the actin cytoskeleton with a cortical ring formation with increased PGE_2_ [114]. As mentioned, we demonstrated increased FFSS in UNX mice and rats [73].

### 2.2. Tensile Stress

The differential pressure (swelling pressure) between the capillary and Bowman space drives filtration while generating circumferential stress (~50 Kilopascals, kPa) and axial stress (0.3 kPa). Being much greater than axial stress, circumferential stress is considered the principal cause of tensile stress (calculated force of 2 nanonewtons) experienced in the podocyte foot process (0.04 µm^2^ area). The circumferential stress was shown to be borne by ≥20 actin filaments/foot process. Further, the capillary is assumed as a nonlinear elastic spring where the axial tension is countered by the elastic modulus of the structural elements of the capillary wall (collagen IV, laminin, etc.) with estimated Young’s modulus of 2000–5000 kPa [115].

Podocytes function as mechanosensors in response to mechanically induced stretch. The tensile stress due to capillary pressure causes podocytes to elongate along the GBM and develop a tensile strain to remain attached. Experimental biaxial stretch (0.5 Hz with 5% linear strain, 3 days) was shown to result in altered gene expression [105], activation of signaling cascades and generation/release of humoral factors and receptors [108,115,116,117].

#### Tensile Stress Alters the Actin Cytoskeleton Organization, Cell Adhesion, and Gene Expression of Podocytes

Capillary stretch targets slit junctions between foot processes [107,112]. Tensile stress has been shown to decrease transverse actin fibers and increase radial fibers in podocytes [115]. It also upregulates the secreted protein acidic and rich in cysteine (SPARC, i.e., Osteonectin) [118] and osteopontin [105,119] that attenuate the effect of cell stretch/detachment. In vitro studies showed that cell stretch induces p21, p38 MAPK, ERK1/2, and JNK [118,119,120,121,122,123,124,125]. We consider increased flow-induced shear as the primary of cellular stress in the early stages. However, increased tensile stress exacerbates the damage and accelerates the loss of function.

## 3. Tubulocentric and Podocentric Effects of Hyperfiltration

Deterioration of podocyte structure and glomerular function are primary and extensively studied features of hyperfiltration-induced changes during the early stages of glomerular disease. Physiological functions of glomerular and tubular segments are interdependent. Thus, both glomerular and tubular segments are subject to the effects of adaptive and maladaptive hyperfiltration and undergo changes that determine the pathogenesis of kidney disease.

### 3.1. Tubular Function and Glomerular Hyperfiltration

Tubular response to hyperfiltration and tubulo–glomerular interdependence are illustrated in several ways. Briefly, (i) tubular and glomerular components closely interact for the tubuloglomerular feedback (TGF) to regulate and maintain renal blood flow, GFR, and the tubular flow rate. [126]. (ii) Primary cilia in tubular cells function as mechano-sensors of hyperfiltration and activators of signaling [127]. (iii) Our collaborators have recently shown that hyperfiltration in unilaterally nephrectomized mice affects MAPK/ERK signaling, suggesting proliferation in principal cells of the collecting duct [128]. (iv) Glucose and sodium reabsorption by sodium-glucose co-transporters (SGLTs) in the proximal tubule increase due to tubular hyperplasia and hypertrophy, which, in turn, activates senescence-associated genes, inflammation and fibrosis [129]. (v) Sodium-glucose cotransporter-1 (SGLT1) activity in macula densa cells releases nitric oxide that promotes early hyperfiltration to recalibrate glucose and sodium levels [28]. (vi) SGLT2 inhibitors (SGLT2i) effectively normalize the tubuloglomerular feedback and lower hyperfiltration through inhibition of sodium and glucose reabsorption [130]. (vii) Persistent hyperfiltration increases tubular reabsorption and the cellular demand for oxygen that exacerbates oxidative stress [28,129]. (viii) The onset of albuminuria indicates either impaired reabsorption or ultrafiltrate protein levels exceeding the reabsorptive capacity for albumin by proximal tubular cells. Albuminuria exacerbates tubular stress [130]. (ix) Several molecules have been suggested as biomarkers of tubular stress and injury. These include N-acetyl-β-D-glucosaminidase, Neutrophil Gelatinase-Associated Lipocalin (NGAL) and Kidney Injury Molecule-1 (KIM1), urinary free Retinol-Binding Protein (RBP) and Cystatin C [131]. (x) Tubulointerstitial oxidative stress, inflammation, hypoxia and fibrosis relate to progression of kidney disease in diabetes [132].

### 3.2. Podocytes and Glomerular Hyperfiltration

Podocytes cover the GBM along the outer aspect of the capillary with a network of interdigitating foot processes in Bowman space. While the glomerular capillary is the site of hemodynamic changes, terminally differentiated and irreplaceable podocytes are immediately affected by hemodynamic changes. Podocytes provide the site of plasma filtration through slit pore junctions formed by their interdigitating foot processes, act as sensors of vascular changes and synthesize components of the glomerular basement membrane (GBM) that covers the capillary [133]. As discussed above, their location exposes podocytes to tensile stretch generated by capillary pressure and to shear stress due to ultrafiltrate flow along their apical, lateral, and basal surfaces [134].

We postulated that FFSS activates initial COX2-PGE_2_-EP_2_ signaling in podocytes. Overall, COX2-PGE_2_-EP_2_ axis with the activation of AKT-GSK3β-β catenin and c-Src-PLD-mTOR signaling mediates the early effects of FFSS [134]. We also demonstrated the ex vivo effect of FFSS on podocytes in intact glomeruli where FFSS or PGE_2_ caused an increase in glomerular albumin permeability of isolated rat glomeruli that was blocked by indomethacin, an inhibitor of prostaglandin synthesis [73]. Additional work using unilaterally nephrectomized sv129 mice that have normal nephron endowment and Os/+ mice that are born with low nephron number showed increased albuminuria and glomerular expression of COX2 and PGE_2_ receptor EP_2_ proteins [73,135].

#### Glomerular Hyperfiltration Results in Podocytes Loss

Overall, hyperfiltration-associated stress induces glomerular hypertrophy. However, podocyte hypertrophy is not in proportion with the increase in the GBM length. Secondly, podocytes respond to stress (e.g., hyperfiltration) by contracting actin fibers, resulting in denudation of the GBM [136]. These disparate changes in GBM and podocytes result in: (i) areas of the GBM left uncovered by podocyte processes adding to podocyte injury, detachment and loss; (ii) adherence of the capillary to parietal epithelium; (iii) formation of synechia and segmental sclerosis [27,113]. Viable podocytes were detected in urine samples following detachment from GBM in patients with FSGS [137], IgA nephropathy [138] and diabetes [139]. Detachment and loss of podocytes exacerbates proteinuria in these conditions.

Cellular adhesion to the extracellular matrix involves membrane glycoproteins and proteoglycans [140]. Podocytes cover the outer aspect of the matrix through interdigitating foot process with slit pore junctions and also contribute to matrix structure by synthesizing its components. We previously showed that FFSS caused derangement of the actin cytoskeleton in podocytes, activated the COX2-PGE_2_-EP_2_ signaling axis and resulted in Akt-glycogen synthase kinase-3β-β-catenin and MAPK/ERK signaling. We recently used IMPres analysis, a novel bioinformatics algorithm for hypotheses generation using pathway networks and multi-omics data [141]. This analysis showed that activation of the COX2, EP_2_, and β-catenin is linked to changes in proteoglycans and galactose metabolism in FFSS-treated podocytes involved in glycocalyx remodeling and cell attachment/detachment [142].

Thus, increased fluid flow during early hyperfiltration affects the size, shape and adhesion of podocytes. Ultrastructural integrity of podocytes is essential for the glomerular filtration barrier function. As mentioned, we have shown that COX2-PGE_2_-EP_2_ axis, β-catenin signaling and glycocalyx remodeling pathways are upregulated in podocytes exposed to fluid flow shear stress. Therefore, we are pursuing the idea that biomechanical stress triggers signaling events that begin at the plasma membrane.

## 4. The Plasma Membrane as the Cellular Point of Contact with Biomechanical Forces

### 4.1. The Plasma Membrane Functions as a Sensor of Mechanical Stress

The asymmetrical plasma membrane (lipid bilayer) is the outermost part of the cell and functions as the first sensor of shear and stretch forces through its glycoproteins and lipids. These components determine membrane characteristics (e.g., porosity, microviscosity, stiffness, contractibility, etc.) and the transmission of force across the membrane and cell–cell junctions. Membrane lipid rafts provide the flexibility to regroup and reorganize molecules for converting a mechanical signal into a chemical signal that acts on downstream cellular organelles and the nucleus through the cytoplasmic milieu [143,144].

Fluid flow on the membrane likely plays a crucial role in cellular mechanotransduction [145,146], and membrane phospholipids are highly responsive to changes in membrane homeostasis and function. Phospholipids function as sources of fatty acids, glycerides and bases that either act directly or form effective mediators through interactions with other molecules. However, the molecular basis of shear-induced mechanochemical signal transduction in podocytes is largely unknown. Recent studies show a decrease in membrane lipid cholesterol and mitochondrial ATP generation in shear-force treated endothelial cells [147].

### 4.2. Membrane Lipid-Bound Fatty Acids Are Precursors of Signaling Mediators

Phospholipids, glycolipids/glycophospholipids and cholesterol are the major membrane lipids. Most cellular cholesterol is localized in the plasma membrane while the endoplasmic reticulum forms complexes with phospholipids and contributes to membrane properties such as fluidity and permeability. Major membrane phospholipids are (1) Glycerophospholipids—phosphatidylcholine (GPCho/PC), phosphatidyl ethanolamine (GPEtn/PE), phosphatidyl serine (PS/PtdSer/PS), phosphatidyl inositol (GPIns/PtdIns/PI), phosphatidic acids (GPA/PA) and lyso-phosphatidic acids (LPA), and cardiolipin (CL); (2) sphingolipids (SP), sphingomyelins (SM) that are a source of ceramides (CE/CER) and (3) gangliosides. Membrane lipids also provide phospholipids/fatty acids for endocannabinoids that are currently being studied extensively concerning their inflammatory and anti-inflammatory effects. Non-membrane lipids such as steroid hormones and platelet activating factor (PAF) are also important for signal transduction.

PC and PE are the most abundant phospholipids in the plasma membrane. PI, PS, PA, lyso-PA, CL, and sphingolipids (sphingomyelin, ceramide, cerebrosides, gangliosides) contribute to a range of membrane properties and organization of signaling domains that are integral to the cellular response to stress. These lipids also function as sources of PUFA, formation of signaling molecules (e.g., diacyl glycerol), formation of microvesicles and formation of lipid rafts. A detailed discussion of these lipids and their roles is beyond the scope of this article.

Membrane phospholipids are virtually the instantaneous source of acyl groups (fatty acids) from sn-1 and sn-2 positions of the glycerol moiety. Relative amounts of ω-6 (n-6) and ω-3 (n-3) polyunsaturated fatty acids (PUFA) are important determinants of membrane properties and physiological functions. Generally, ω-3 PUFAs are considered sources of anti-inflammatory and antioxidant molecules while excessive ω-6 PUFA generate pro-inflammatory products. A very high ratio of ω-6:ω-3 PUFA (~15:1) in Western diets has been correlated with chronic diseases, and a balanced (lower) ω-6/ω-3 ratio (≤4:1) is considered healthy [148,149].

### 4.3. Phospholipases Release Fatty Acids from Membrane Phospholipids

Phospholipid-bound fatty acids are released by phospholipases that catalyze hydrolysis at different positions in the molecule. Briefly, phospholipase A1 (PLA1) cleaves the acyl ester bond (generally a saturated fatty acid) from sn-1 position; numerous PLA2 can act on sn-2 position fatty acid (typically an unsaturated fatty acid); phospholipase B (lysophospholipase) removes both sn-1 and sn-2 fatty acids; phospholipase C activity cleaves the glycerophosphate bond releasing diacyl glycerol and phosphorylated base; and phospholipase D cleaves after the phosphate group to release phosphatidic acid and the polar head group [150,151,152]. Omega-6 and ω-3 polyunsaturated fatty acids released by phospholipase activity are metabolized to generate oxidized products (oxylipins) through pathways involving at least one step of dioxygen-dependent oxidation [153]. Thus, ω-3 and ω6 PUFA generate several oxylipins that modulate metabolic homeostasis as well as disease-associated inflammation.

#### Mechanical Stress Activates Phospholipases

Fluid flow shear stress upregulates c-Src phosphorylation, phospholipase D (PLD) activation and mammalian target of rapamycin (mTOR) signaling in podocytes [154]. Mechanical (magnetic) force applied to cultured podocytes was shown to recruit Rho and filamentous actin (F-actin) and stimulate phospholipase D (PLD). PLD is a downstream effector of Rho, and its activation involves Gα12/13/Rho/F-actin and calmodulin-dependent kinase [155]. Shear-induced Piezo1 was found to activate PLA2 causing intracellular calcium release leading to TRPV4 channel opening, resulting in prolonged calcium elevation and cytopathological events in endothelial cells [156].

## 5. Arachidonic Acid (ω-6 PUFA) Generates Lipid Mediators through the Cyclooxygenase, Lipoxygenase and Cyto-Chrome P450 Pathways

**Figure 2:**
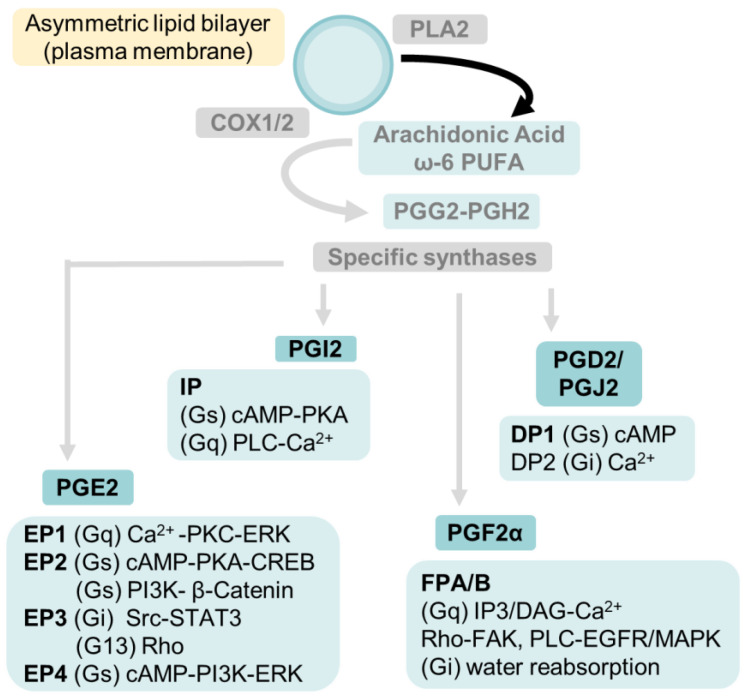
**Key steps in the synthesis of prostaglandins from membrane phospholipid derived arachidonic acid.** Phospholipase A2 activity releases free ω-6 PUFA arachidonic acid (ARA) from phospholipids in the asymmetric plasma membrane. COX1/2 converts free ARA into PGG_2_/PGH_2_ utilized by specific synthases to form prostanoids—prostaglandins E2, I2, J2, D2, F2α and thromboxane A2 (shown separately). Each of these bioactive metabolites binds to specific G-protein-coupled receptors for signaling. Receptors, specific G proteins (in parentheses) and activated the signaling pathways in a separate box under each prostaglandin. PGI_2_, an active metabolite, exists transiently and is metabolized to 6-keto-PGF1α (not shown here).

**Figure 3:**
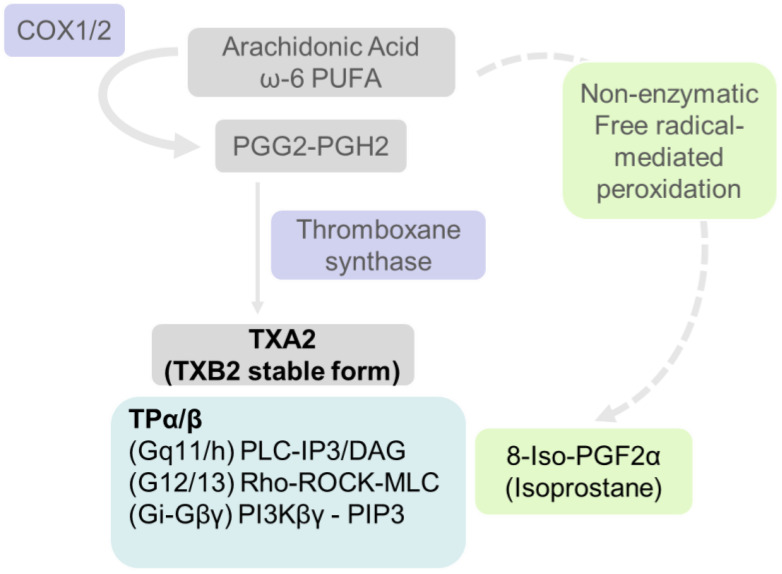
**Outline of thromboxane synthesis and signaling.** TXA_2_ is generated from free arachidonic acid through COX1/2-catalyzed reactions followed by thromboxane synthase activity as shown in Figure 2. A separate box under TXA_2_ shows its receptor TPα/β, a GPCR. TXA_2_ receptor activates signaling pathways according to the G protein involved as indicated. Isoprostane, 8-iso-prostaglandin F_2__α_ (8-iso PGF_2__α_), is formed by peroxidation of arachidonic acid and is another ligand for thromboxane receptor.

**Figure 4:**
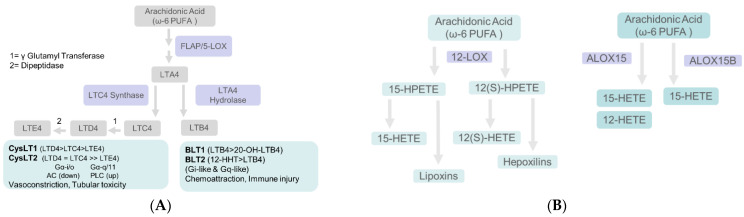
(**A**) **Schematic showing leukotrienes synthesis and signaling (LOX-5 pathway).** Free arachidonic acid (Figure 2) is converted into LTA_4_ by 5-LOX by activating protein (FLAP). LTA_4_ is converted into LTB_4_ by LTA_4_ hydrolase or LTC_4_ by LTC_4_ synthase. Gamma-Glutamyl Transferase attaches glutathione to LTC_4_ and generates LTD_4_. A dipeptidase activity converts LTD_4_ into LTE_4_. Box under LTB_4_ shows its G-protein-coupled receptors BLT1 and BLT2 and ligand preferences and LTB_4_ functions. The text box under cysteinyl leukotrienes LTC_4_, LYD_4_ and LTE_4_ indicates receptors CysLT_1_ and CysLT_2_ with ligand preferences. Downregulation (down) of adenyl cyclase (AC) upregulation (up) of phospholipase C (PLC) coupled to specific G proteins is indicated followed by the cellular effects of cysteinyl LTs. (**B**). **LOX-12 and LOX-15 pathways:** Left: Arachidonic acid is metabolized by 12-LOX to 15-hydroperoxyeicosatetraenoic acid/arachidonic acid 15-hydroperoxide (15-HPETE/15-HpETE) or 12(S)-HPETE/12-HPETE. 15-HPETE is further metabolized to yield 15-HETE and lipoxins (specialized pro-resolving mediators). 12-HPETE generates 12-HETE and hepoxilins (anti-inflammatory). Right: Lipoygenase-15 (ALOX15) and ALOX15B generate 15-HETE as the dominant product with a small amount of 12-HETE by ALOX15 activity.

**Figure 5:**
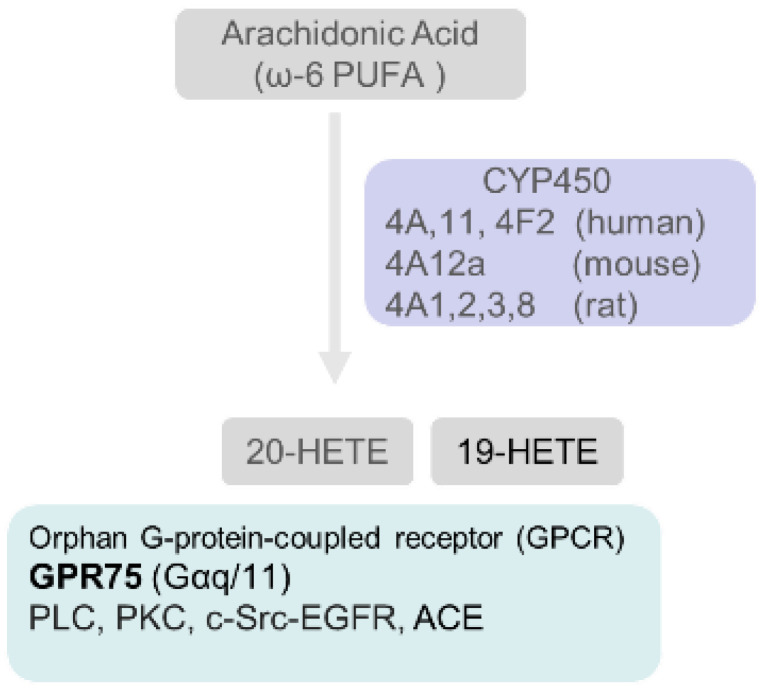
**Synthesis, receptor and signaling of 20-HETE.** Arachidonic metabolism by CYP450 hydroxylases in different species (shown: human, mouse and rat) convert arachidonic acid into 20-HETE (and 19-HETE). 20-HETE is a ligand of G-protein-coupled receptor GPR75 and activates PLC, PKC, c-Src-EGFR, ACE leading to vasoconstrictive and natriuretic effects. 20-HETE can be further metabolized by CYP-epoxygenases, LOX and COX. UDP-glucuronosyltransferases metabolizes 20-HETE to form glucuronides in humans.

**Figure 6:**
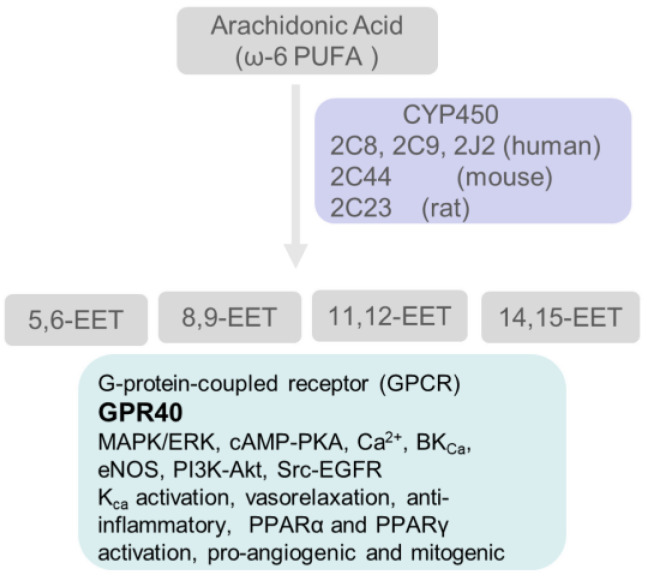
**Synthesis, receptor, and signaling of EETs.** Arachidonic acid is converted into four EET regioisomers by species-specific CYP450 epoxygenases (human, mouse, and rat isoforms shown). GPR40, shown in the box under EETs, is a GPCR activated by EETs, resulting in activation of signaling pathways and cellular effects of EETs. EETs are metabolized by soluble epoxide hydrolase (sEH) to form corresponding dihydroxyeicosatrienoic acids (DHET) with much lower activity.

Membrane phospholipid derived arachidonic acid (ARA; 20:4 ω-6) and its dietary precursor linoleic acid (LA; 18:2 ω-6) are the main ω-6 PUFA. The linoleic acid status reflects recent dietary fatty acid intake. Its excess worsens kidney pathology and may predict the risk for CVD and mortality [157]. Lipid-bound arachidonic acid is released by phospholipase activity and metabolized by cyclooxygenases 1 and 2 (COX1, COX2, also known as prostaglandin H synthase), lipoxygenases (LOX) and cytochrome P450 (CYP2 epoxygenases and CYP4 ω-hydroxylases). As outlined in Figure 2, Figure 3, Figure 4, Figure 5 and Figure 6, the oxylipins derived from arachidonic acid (eicosanoids) include prostanoids (prostaglandins, prostacyclins and thromboxanes), leukotrienes, epoxyeicosatrienoic acids (EETs) and hydroxyeicosatetraenoic acids (HETEs).

### 5.1. Cyclooxygenases Catalyze the Conversion of Arachidonic Acid to Prostaglandins and Thromboxane

Figure 2 outlines the cyclooxygenase-catalyzed synthesis of intermediates PGH_2_/PGG_2_ Prostaglandin (PG) E_2_ (PGE_2_), prostacyclin (PGI_2_), prostaglandin D_2_ (PGD_2_), and prostaglandin F_2α_ (PGF_2α_). Specific receptors and major signaling pathways activated by these mediators are also indicated. COX1 and COX2 each generates prostaglandins in a cell- and environment-specific manner. Isoprostanes are proinflammatory derivatives of arachidonic acid through non-enzymatic peroxidation by reactive oxygen species. The overall contribution of these enzymes to kidney pathophysiology appears to depend on the net balance of oxylipins and may be protective or harmful.

Glomerular synthesis of prostaglandins was demonstrated using isolated rat glomeruli [158]. PGF_2α_ and PGJ_2_ are derived from PGH_2_ (Figure 2). A slight increase in urinary PGF_2α_ was detected in patients at an early stage of nephropathy. However, a significant decrease in urinary PGF_2α_ was observed during end-stage renal disease [159]. Infusion studies in dogs showed a natriuretic effect of PGF_2α_ through inhibition of salt reabsorption at the thick ascending limb of Henle’s loop [160].

PGD_2_ was shown to have renoprotective and antifibrotic effect in a rat model of hypertension and is considered an early marker of renal injury [161]. Its antifibrotic effects were shown to be through inhibition of TGFβ [162]. PGD_2_ is converted non-enzymatically to PGJ_2_ and 15-deoxy-Δ 12,14-prostaglandin J_2_ (15d-PGJ_2_). PGD_2_ and PGJ_2_ are ligands of DP2 and DP1, G-protein-coupled receptors (Figure 2). DP2 activation downregulates cAMP and upregulates PGD_2_, while activation of DP1 upregulates cAMP. PGD_2_ and PGJ_2_ activate PPARγ and have been shown to have a role in the inhibition of pro-inflammatory cytokines and transcription factors and vascular remodeling and renoprotection in ischemia reperfusion injury [163,164].

#### 5.1.1. COX2 Expression Is Upregulated in Podocytes Exposed to FFSS

Fluid flow shear stress or mechanical stretch alters COX expression in podocytes: Podocytes express both COX1 and COX2 [114,165,166] and shear stress-treated podocytes show upregulation of COX2 but no change in COX1 expression [73,114,135]. Previous studies showed upregulation of COX2 and PGE_2_ in podocytes after mechanical stretch [120]. It is noteworthy that while basal expression of COX2 is required for podocyte survival, overexpression increases susceptibility to cellular injury. Injury may occur, in part, due to thromboxane and its receptor [167,168].

#### 5.1.2. Arachidonic Acid Metabolite Prostaglandin E_2_ (PGE_2_) Is a Major Mediator of Biomechanical Stress

PGE_2_ is the most abundant prostanoid under physiological conditions and the subject of many studies. PGE_2_ is synthesized from endoperoxide (PGH_2_) by Prostaglandin E_2_ synthase (PGES, 3 isoforms) [169]. PGE_2_ is a ligand of four E-prostanoid (EP_1–4_) receptors [170,171] expressed in a cell/nephron segment-specific manner. Each G-protein-coupled receptor activates the signaling pathway indicated (Figure 2).

5.1.2a. Biomechanical stress, PGE_2_ and podocytes: PGE_2_ modulates multiple processes through its receptors. Deficiency of EP_4_, EP_2_, or EP_1_ resulted in smaller glomeruli suggesting an essential role of PGE_2_ during development [172]. Mouse podocytes express EP_1_, EP_2_, and EP_4_ [73] with EP_4_ expression > EP_2_ under basal conditions [73,173]. In vitro studies using podocytes and in vivo studies using mouse models led us to postulate the COX2-PGE_2_-EP_2_ axis as a mediator of shear-induced stress in podocytes [114]. Our results also showed FFSS-induced upregulation of EP_2_ expression but not EP_4_ [73]; EP_4_ was previously shown upregulated by tensile stress [120]. A recent report also described the upregulation of COX2 in podocytes treated with PGE_2_ [174]. We recently described that PGE_2_-induced activation of EP_2_ led to upregulation of AKT-GSK3β-β catenin signaling. These studies identified the transcription factor β-catenin as a key mediator of FFSS-induced changes [175].

5.1.2b. Biomechanical stress, PGE_2_ and tubular cells: EP_1_ expression in collecting ducts, and EP_3_ in the tubular segment in the outer medulla and cortex have been demonstrated [176,177]. FFSS- and stretch-induced tensile stress affect intracellular signaling in tubular cells in a segment-specific manner [127,178] as shown in studies using principal and intercalated cells of renal cortical collecting duct [179,180]. Increased flow in the cortical collecting duct increased sodium reabsorption and potassium secretion into urine [181]. Enhanced sodium transport due to hyperfiltration in UNX mice was associated with ERK stimulation [128]. Thus, PGE_2_ receptors in various tubular segments respond to biomechanical stress.

Tensile stress and FFSS have been shown to cause opposite effects on PGE_2_ release from distal tublar cells. Experimental tensile stress by circumferential stretch resulted in decreased PGE_2_ secretion, while FFSS using laminar flow resulted in increased release of PGE_2_ from cultured tubular cells. FFSS also upregulated COX2, neutral sphingomyelinase, endothelin, phospho-ERK and phospho-p38 [177,179,182,183,184]. In proximal tubular cells, cyclical stretch caused ERK-dependent release of arachidonic acid [185] while FFSS resulted in cytoskeletal re-organization through redistribution of stress fibers from the basolateral to apical surface with new apical junctional complexes [186,187]. These studies show differences in the effects of stress due to fluid flow shear and stretch. Beta-catenin in podocytes and p38 MAPK-ERK in tubular epithelial cells appear to be key mediators of downstream signaling. Thus, podocytes and tubular cells utilize PGE_2_ for transducing the effects of shear stress and tensile stress through COX2-PGE_2_.

#### 5.1.3. Arachidonic Acid Metabolite Prostacyclin (PGI_2_) and Biomechanical Stress

PGI_2_ is synthesized from COX-derived endoperoxide intermediate catalyzed by prostacyclin synthase (CYP8A) (Figure 2). Prostacyclin binds to a G(Gi, Gq, Gs)-protein coupled receptor of the rhodopsin-type receptors superfamily [188] and activates multiple signaling pathways. A cAMP-PGI-PPARδ (peroxisome proliferator-activated receptors-delta) axis is believed to be the principal signaling mechanism for PGI_2_ [189]. One limitation on the use of PGI_2_ is its rapid metabolism to a nearly inactive product, 6-keto-PGF1α (not shown in Figure 2). Several new stable analogs of PGI_2_ include cicaprost (CCP, a highly selective IP agonist), iloprost (ILP, less selective), beraprost sodium, UT-15, and taprostene are now available to target the receptor [190].

5.1.3a. Role of PGI_2_ in hyperfiltration: Prostacyclin is a vasodilatory prostanoid involved in maintaining renal blood flow and GFR, and attenuating the effects of systemic vasoconstriction [190,191,192]. PGI_2_ actions include inhibition of platelet aggregation and inhibition of vascular smooth muscle cell proliferation. PGI_2_, tubuloglomerular feedback inhibitio, and increased glucagon secretion mediate hyperfiltration and increased glomerular permeability associated with high dietary proteins of animal origin [193,194].

#### 5.1.4. Thromboxane A_2_ (TXA_2_) Is Associated with Inflammation during Hyperfiltration

Thromboxane A_2_ (TXA_2_) synthesis is catalyzed by thromboxane A_2_ synthase (TXA_2_S) from prostaglandin endoperoxide (PGH_2_) generated by COX activity on free arachidonic acid (summarized in Figure 3). TXA_2_ is detected in its stable form TXB_2_. TXA_2_ activates thromboxane receptors (TPα and TPβ) that are G (Gq and Gs)-protein-coupled receptors expressed in podocytes [195,196]. TXA_2_ signaling results in phospholipase C activation and RhoGEF activation, causing PI3K-mediated calcium release, cAMP generation and cytoskeletal changes [197]. TXA_2_ is a pro-inflammatory vasoconstrictor and facilitator of platelet aggregation. It regulates renal hemodynamics and water and electrolyte metabolism and plays a role in the pathophysiology of renal diseases including diabetic nephropathy. A balance between the levels of TXA_2_ and PGI_2_ is considered essential for renal homeostasis and regulation of platelet aggregation.

5.1.4a. Thromboxane levels are elevated during early and late diabetic changes: Increased synthesis of PGE_2_, 6-keto-PGF_1α_ vs. TXB_2_ was noted in glomeruli from rats at early-stage diabetes (9–15 days post-STZ injection). However, only TXB_2_ was increased at 25–28 days post-STZ injection suggesting other sources of TXB_2_ (e.g., platelets) or activation of thromboxane synthase at later stages after STZ treatment [198]. Another study reported that renal mass ablation (>70%) resulted in increased urinary TXB_2,_ suggesting a key role of platelet aggregation and intraglomerular thrombosis in the development of glomerulosclerosis in this model [199].

### 5.2. Lipoxygenases Catalyze the Conversion of Arachidonic Acid to Leukotrienes (LT) and Hydroxyeicosatetraenoic Acids (HETE)

Arachidonic acid oxidation catalyzed by 5-lipoxygenase (5-LOX) initiates reactions leading to four stable leukotrienes (LT), namely LTB_4_ and three cysteine-containing LTC_4_, LTD_4_ and LTE_4_. Leukotrienes bind to and activate G(Gq/Gi) protein-coupled receptors (Figure 4A). Montelukast, an antagonist of the leukotriene receptor, was shown to block the IL-13-induced internalization and downregulation of ZO-1 protein in podocytes [200]. Gq-dependent signaling in podocytes was found to upregulate COX2 expression and PGE_2_ production [195]. Arachidonic acid is also utilized by (i) 12-LOX to generate 12/15-hydroxyeicosatetraenoic acids and lipoxins (a specialized pro-resolving mediator) and by (ii) 15-LOX to generate 15/12-HETE (Figure 4B).

#### 5.2.1. Leukotrienes Mediate Glomerular Injury

Direct role of LTs in hyperfiltration-induced changes is not yet described. However, LTs mediate glomerular and tubular injury in several conditions, including diet-induced lipidemia and glomerular sclerosis [201,202]. Since both COX and LOXs act on arachidonic acid, the ‘COX to LOX’ shunting may contribute to nephrotic syndrome [203]. LTB_4_ is a chemoattractant and mediates recruitment of infiltrating polymorphonuclear cells which, in turn, exacerbate the immune-mediated glomerular injury and impair renal function [204]. Cysteinyl leukotrienes are vasoconstrictive and cause lower renal blood flow and GFR [205]. LTB_4_ and cysteinyl leukotrienes were shown to enhance allograft rejection [206].

#### 5.2.2. Urinary Leukotriene Metabolites Indicate Tubular Injury

Increased LTC_4_ production induced by cytotoxic drugs resulted in nuclear accumulation of NADPH oxidase, causing hydrogen peroxide-mediated oxidative DNA damage and cell death [207]. Increased Cysteinyl LTs and other Cysteinyl-X conjugates are acetylated by acetyltransferase 8 (NAT8) for excretion, thus preventing tubular toxicity. Urinary N-Acetyl-LTE_4_ has been proposed as a useful marker of renal tubular damage [207,208].

### 5.3. Cytochrome P450 Enzymes Catalyze the Conversion of Arachidonic Acid to Hydroxy- and Epoxy-Oxylipins

In addition to cyclooxygenases and lipoxygenases, enzymes of the cytochrome P450 (CYP450) superfamily also metabolize arachidonic acid to form effective modulators of kidney function (Figure 5). Specifically, enzymes of the CYP P450 4A and 4F families (human) catalyze the formation of 20-hydroxyeicosatetraenoic acid (20-HETE), and enzymes of the CYP2C and CYP2J families catalyze the synthesis of four epoxyeicosatrienoic acids (EETs).

#### 5.3.1. 20-Hydroxyeicosatetraenoic Acid (20-HETE)

20-HETE is the primary product of CYP450A and CYP4F activities. 20-HETE uses a recently identified GPR-75, a G (Gαq/11)-protein-coupled receptor that activates inositol phosphate production. GPR75 interaction with GPCR kinase-1 leads to c-Src-mediated activation of EGFR, upregulation of angiotensin-converting enzyme (ACE) and endothelial dysfunction [209]. 20-HETE can be metabolized by cyclooxygenase to form 20-OH-PGE_2_ that promotes lipid accumulation in mesenchymal stem cell-derived adipocytes resulting in upregulation of adipogenic regulators PPARγ and β-catenin [210,211].

5.3.1a. Role of 20-HETE in hypertension: Overall, 20-HETE is currently thought to have opposing effects. Vasoconstriction shows its pro-hypertensive effect through increasing myogenic tone, tubuloglomerular feedback, smooth muscle cell migration and proliferation (vascular remodeling), endothelial dysfunction, ACE expression and inflammation. In contrast, it increases natriuresis by inhibiting tubular reabsorption of sodium as an antihypertensive agent [212,213]. Thus, high levels of vascular 20-HETE or decreased synthesis of tubular 20-HETE may lead to hypertension.

5.3.1b. Direct effect of 20-HETE podocytes: We demonstrated that 20-HETE preserves the glomerular filtration function against the injury caused by puromycin aminonucleoside (PAN) and ethanol [214,215]. These results suggest a direct effect of 20-HETE on podocytes.

#### 5.3.2. Epoxyeicosatrienoic Acids (EETs)

Four EET regioisomers (14,15-EET, 11,12-EET, 8,9-EET, and 5,6-EET) are synthesized from arachidonic acid by P450 epoxygenases of CYP2C and CYP2J families (Figure 6). EETs are metabolized by a soluble epoxide hydrolase (sEH) that converts epoxy fatty acids into corresponding vicinal diols (dihydroxyeicosatrienoic acids, DHET) with far less activity than epoxides. Recent studies demonstrated that three EETs (8,9-EET, 5,6-EET, 11,12-EET but not 14,15-EET) could be further metabolized by COX [216,217].

GPR40 was recently reported as a low-affinity G-protein-coupled receptor of EETs that binds to receptor agonist (GW9508) and EETs in the order GW9508 > Arachidonic acid > 11,12-EET > 14,15-EET > 8,9-EET, 5,6-EET, and 11,12- and 14,15-dihydroxyeicosatrienoic acids (products of epoxide hydrolysis). Activation of GPR40 by 11,12-EET upregulated connexin-43 (Cx43) and COX2 expression in endothelial cells via MAPK-ERK phosphorylation [218].

5.3.2a. EETs protect renal and cardiovascular function: EETs activate ion channels (TRPV4, BK_Ca_) and signaling pathways, and induce anti-inflammatory, anti-fibrotic, and anti-apoptotic changes. These include vasodilation, angiogenesis and cardioprotection against ischemia-reperfusion injury [219]. EETs lower blood pressure through their effects on vascular endothelial cells and epithelial transport. These effects attenuate the progression of kidney and cardiovascular diseases [220]. Early-stage diabetes in a rat model was shown to lower EETs and 20-HETE but increase TGF-β1 in glomeruli [221]. EET hydrolysis increases in early-stage kidney disease. Tissue sEH expression is increased in rodent models of obesity, diabetes and cardiovascular disease. Non-vasomotor regulatory effects of EETs include anti-inflammatory and anti-atherogenic effects, stimulation of lipid metabolism and regulation of insulin sensitivity.

5.3.2b. Regioisomer-specific effects of EETs: Based on the similarities in their effects, EETs are often treated as one group. However, EET regioisomers may cause quantitatively and even qualitatively different effects [222]. For example, (i) showed that only exogenous 8,9-EET dose-dependently protected glomerular function indicating its direct effect on podocytes [223]. (ii) While 11,12-EET and 14,15-EET have been used for most studies on cardiovascular and renal functions [224,225], 14,15-EET is the best substrate for sEH [226,227] and only 11,12-EET inhibits basolateral K^+^ channels in the renal cortical collecting duct [228].

## 6. EPA and DHA (ω-3 PUFA) Generate Lipid Mediators through the Cyclooxygenase, Lipoxygenase and Cytochrome P450 Pathways

Omega-3 PUFA are integral to membrane lipids along with ω-6 PUFA. The 18-carbon chain α-linolenic acid (ALA; 18:3 ω-3) and its elongation and desaturation products eicosapentaenoic acid (EPA; 20:5 ω-3), and docosahexaenoic acid (DHA; 22:6 ω-3) are the main dietary ω-3 PUFA. Modern ‘Western diet’ contains ω-6 and ω-3 PUFA at a 10–15:1 (ω-6:ω-3) ratio that is considered too high and proinflammatory while a 4:1 to 2:1 ratio has been found to lower inflammation, and decrease the risk of several cancers (e.g., renal, breast, prostate) and of rheumatoid arthritis [229,230]. A balance between ω-3 and ω-6 PUFA appears to favor homeostasis. A reciprocal relationship between levels of ω -6 and ω-3 fatty acids indicates competition for binding to membrane lipids. We speculate that increasing levels of ω-3 PUFA protect against hyperfiltration-induced injury. Therefore, we included the following brief discussion on ω-3 PUFA despite a lack of extensive information.

Biological actions of ω-3 PUFA include regulation of vasomotor tone and renal sodium excretion. Ω-3 PUFA cause decreased levels of triglycerides and vasoconstricting eicosanoids, down-regulation of ACE activity, decreased formation of ANG II levels, lower expression of TGF-β, improved generation of endothelial NO, stimulation of parasympathetic nervous system in both normotensive and hypertensive, dyslipidemic, diabetics and older subjects [231,232,233,234]. However, the effect of ω-3 PUFA may also depend on the amount of PUFA. Thus, large quantities of ω-3 PUFA may elicit oxidative stress indicated by 4-hydroxy hexenal (4-HHE, a lipid peroxidation product), and upregulated p38 kinase in aortic tissue endothelial cells [235].

### 6.1. Protective Effects of ω-3 PUFA against Glomerular and Kidney Injury

Fish oil (ω-3 PUFA) was found to have anti-inflammatory, anti-proteinuric and renoprotective effects in diabetes [236,237], cyclosporine-induced nephrotoxicity, lupus nephritis and IgA nephropathy [238,239]. We have also observed a protective effect of ω-3 PUFA on glomerular function [unpublished data].

### 6.2. Protective Effects of ω-3 PUFA Metabolites

Enzymes of COX, LOX and CYP450 pathways also act on ω-3 PUFA (EPA and DHA, i.e., products of α-linolenic acid) to generate parallel oxylipins [240]. Omega-3 PUFA (DHA, EPA) are utilized with equal or better efficiency by the enzymes that metabolize ω-6 arachidonic acid [241,242,243]. Systematically organized comprehensive structure-function understanding of these metabolites is not available, but lipidomics data are adding to a better understanding of the significance of ω-3 oxylipins [153,244,245].

## 7. Both ω-6 and ω-3 PUFA Yield Endocannabinoids

Endogenous (Endo-) cannabinoids (EC) are closely related to membrane lipids and eicosanoids through ω-6 and ω-3 PUFA. Arachidonoylethanolamide (AEA, anandamide), 2-arachidonoylglycerol (2-AG), and 2-arachidonylglyceryl ether (2-AGE) synthesized from arachidonic acid are the most studied molecules in this category [246,247]. EC derived from ω-3 PUFA (ω-3 EC) include docosahexaenoyl ethanolamide (DHA-EA, i.e., synaptamide), docosahexaenoyl glycerol (DHG), eicosapentaenoyl ethanolamide (EPA-EA), eicosapentanoyl glycerol (EPG). Ω-3 PUFA also form metabolites with serotonin (DHA-5HT) [248,249]. Cannabinoid receptors CB1R and CB2R are GPCRs that activate adenyl cyclase and MAPK signaling. Relative abundance of CB1R and CB1R is tissue/cell-specific, and both modulate the innate immune response through regulation of cytokines levels. CB1R and CB2R are expressed in glomerular mesangial cells and podocytes.

Activation of peripheral CB1R contributes to glomerular injury, inflammation, lower GFR, albuminuria, loss of renal function and renal fibrosis [250,251]. CB1R activation also modulates the expression, translocation, and activity of SGLT2 in proximal tubular cells that contributes to the development of diabetic nephropathy [252]. CB1R gene deletion, CB1R siRNA or CB1R antagonists (AM6545, SR141716, given at pre-diabetic stage) was shown to attenuate the indicators of nephropathy [253,254,255,256].

In contrast, CB2R-mediated signaling and levels of its ligand (2-AG) are lower in diabetic kidney tissue and cultured podocytes after mechanical stretch [257,258]. The absence of CB2R in glomerular cells worsened renal functional and structural abnormalities in diabetic nephropathy [258]. CB2R agonists have been shown to ameliorate albuminuria, reduce C-C motif Chemokine Receptor-2 (CCR2) expression in the renal cortex and cultured podocytes, lower mesangial expansion and decrease MCP1 expression in animal models of diabetes and obesity [257,259].

## 8. Both ω-6 and ω-3 PUFA Generate Specialized Pro-Resolving Mediators (SPM)

SPMs are relatively recently discovered classes of PUFA derivatives currently being studied for their ability to resolve inflammation. Some SPM have shown renoprotective effects and detailed information on their role in hyperfiltration-induced injury will likely emerge in the future. A brief mention of these compounds is essential given their promising renoprotective effects. Both ω-6 and ω-3 PUFA serve as precursors of endogenous specialized pro-resolving lipid mediators (SPMs) [260,261]. Lipoxins are generated from arachidonic acid through actions of 5-lipoxygenase followed by 12/15-LOX or acetylated COX [262,263].

Resolvins (Rv) are among the most notable lipid mediators for on-site resolution of inflammation and for maintaining homeostasis. EPA-derived RvE are formed by aspirin-modified COX2 and are called aspirin-triggered resolvin (AT-RvDs). A cytochrome P450 enzyme(s) is also believed to participate in the generation of AT-RvEs [264,265]. DHA-derived resolvins are named RvD and RvD1 have been shown to be renoprotective [266,267].

Protectins (PD) are SPM-derived from ω-3 PUFA docosahexaenoic acid (DHA) and n-3 docosapentaenoic acid (n-3 DPA) [268]. Protectin/neuroprotectin D1 (PD1/NPD1) have been found to have protective effects on kidneys and liver [269].

Maresins (MaR) are a new family of anti-inflammatory and pro-resolving lipid mediators which are biosynthesized from docosahexaenoic acid (DHA) with anti-inflammatory and antifibrotic effects of maresins may attenuate effects of diabetic nephropathy [270,271].

## 9. Lipids and Fatty Acids as Biomarkers of Hyperfiltration-The Early Stage of Renal Dysfunction

Hyperfiltration during pre-/early diabetes induces changes in membrane composition and properties. Lipids are potential biomarkers of early changes in plasma membrane. Higher serum levels of sphingomyelin (SM, C18:1) and phosphatidylcholine diacyl (PCaa, C38:0) were associated with higher odds of CKD in pre-diabetic/hyperglycemic individuals. Additional work using db/db mouse model suggested sphingomyelin, C18:1, C38:0 and arachidonic acid in PC as early biomarkers of (pre)diabetes [272].

Leukotrienes (LTs) are strong chemoattractants of immune cells causing oxidative damage in models of diet-induced obesity, nephrotic syndrome and unilateral nephrectomy. Urinary levels of acetylated LTE_4_ are associated with tubular stress and cell death in CKD. Thus, urinary levels of acetylated leukotrienes LTE_4_ may be useful indicators of tubular dysfunction [273,274].

## 10. Current and Evolving Treatments to Modulate Hyperfiltration

Traditional treatment regimens of CKD do not target hyperfiltration for treatment. An important consideration in this regard is that while hyperfiltration is an early event, current treatments aim at established pathophysiology and organ damage. Recently introduced drugs and test substances have been found to modulate hyperfiltration. The following provides an overview of established treatments, recently introduced drugs, and novel compounds.

### 10.1. The Renin-Angiotensin-Aldosterone System (RAAS)

Angiotensin converting enzyme inhibitors (ACEi) and receptor blockers (ARBs) are used for treating proteinuria and hypertension. These drugs decrease glomerular capillary pressure, induce glomerular basement membrane (GBM) remodeling, improve glomerular pore-selectivity and decrease TGF-β, thus decreasing mesangial proliferation and albuminuria. Mineralocorticoid receptor antagonists decrease cardiovascular events in pre-dialysis or on-dialysis patients with CKD [275,276]. RAAS antagonists exert a favorable effect on hyperfiltration but other hypertensive agents such as dihydropyridine calcium-channel blockers and vasodilators are not highly effective on renal autoregulation. RAAS blockade does not completely attenuate hyperfiltration or diabetic kidney injury [277]. Further, RAAS antagonists are not highly effective for treating children born with solitary kidneys who develop hyperfiltration at a very early age and CKD by late adolescence [16,17,278,279].

### 10.2. Sodium–Glucose Transport Protein 2 (SGLT2) Inhibitors

SGLT2 inhibitors (SGLT2i) inhibit glucose/sodium reabsorption in the luminal membrane of the proximal convoluted tubule at filtered glucose levels >80 g/day accompanied by natriuria that facilitates lowering of systemic pressure and hyperfiltration. Increased sodium delivery to the macula densa leads to activation of tubuloglomerular feedback (tubular back pressure) and afferent vasoconstriction. These changes do not interfere with the secretion, levels, or functions of insulin nor with the SGLT1 receptor in the lower proximal tubular area.

SGLT2i are beneficial for normotensive, normoalbuminuric subjects with type 2 diabetes as well as those with CKD but without diabetes [277,280,281,282,283,284]. The beneficial effect of SLGLT2i on non-diabetic kidney disease (higher SNGFR and albuminuria) slows the progression of CKD and lowers the incidence of cardiovascular disease [285,286]. SGLT2i reportedly also improve renal oxygenation and inflammation, improve adipose tissue function and decrease serum leptin, TNF-α and IL-6 while increasing adiponectin [287,288,289]. Ongoing research on widely used SGLT2 inhibitors has highlighted a close interaction between glomerular and tubular compartments, and the significance of tubular oxygenation for glucose reabsorption [28,290]. A notable finding is that these drugs cause an acute decline in glomerular filtration rate (GFR) due to activation of the tubuloglomerular feedback at the beginning of treatment [291]. This decline is offset by delayed decline in GFR over time.

### 10.3. Agonists and Antagonists of Prostaglandin E_2_ Receptors EP_2_, EP4

Prostaglandin E_2_ (PGE_2_), the most abundant product of arachidonic acid metabolism, binds to four receptors (EP_1–4_). EP_2_ antagonist PF-04418948 [292,293] and EP_4_ antagonist ONO-AE3–208 [294,295] were recently used to demonstrate that both EP_2_ and EP_4_ mediate the PGE_2_-induced increase in cAMP [174]. Preliminary work in our laboratory indicates that a combination of receptor agonists and antagonists may provide better attenuation of the effects of hyperfiltration (Unpublished results).

### 10.4. Compounds to Target the Cytochrome P450 Pathway

The CYP450 pathway has been investigated for its renoprotective effect [296]. We reported a direct protective effect of 20-HETE and 8,9-EET or its analog on the glomerular filtration barrier function [214,223]. Soluble epoxide hydrolase (sEH) is a target for treating diabetic nephropathy and hypertension-mediated renal damage [225,297,298,299]. Additionally, inhibition of sEH, deletion of sEH gene (Ephx2) or treatment with EETs or EET analogs enhanced islet cells function and insulin sensitivity [300].

### 10.5. Novel Agonists of Peroxisome Proliferator-Activated Receptors (PPARs)

Receptor isoforms PPARα, PPARδ and PPARγ, PPARs function as unique transcription factors in fatty acid metabolism, energy metabolism and the immune system. PPARs mediate anti-inflammatory effects through inactivation or ubiquitination of NFκb [301]. Agonists of these receptors induce beneficial changes in metabolic diseases. Thus, the PPARα agonists (fibrates) are valuable for treating hypertriglyceridemia; PPARγ agonists (thiazolidinediones TZD) have therapeutic use in type 2 diabetes; and PPARδ activators upregulate mitochondrial function and energy metabolism [302,303,304].

PPARγ is abundantly expressed in podocytes, mesangial cells, vascular endothelial cells and collecting duct cells [305,306]. PPARγ agonist pioglitazone was shown to lower proteinuria, improve GFR, decrease sclerosis and alleviate cell senescence through upregulation of klotho, attenuation of oxidative stress and decreased mitochondrial injury [307]. However, the use of PPARγ agonists is evolving with caution as these drugs may induce fluid retention and peripheral edema, and promote anemia or cause hepatic steatofibrosis, and liver cancer [308].

### 10.6. Lifestyle and Dietary Changes, Low Protein Diets, Plant-Based Diets, ω-3 PUFA-Rich Diets

Hyperfiltration and oxidative damage to proteins and lipids (lipid peroxidation) associated with obesity and/or diabetes precede structural changes in the glomerulus and kidney. Thus, high dietary antioxidants in combination with glycemic and lipid control are considered valuable for preventing organ damage through preserving glomerular endothelial cell permeability [309,310].

#### 10.6.1. Plant-Based Low-Protein Diets

Replacing red meat with plant-derived proteins was shown to lower the risk of CKD [311,312]. Low-protein diets were also found to lower glomerular hyperfiltration, uremic toxins, total acid load, phosphorus and sodium. Recent reports suggest that the renoprotective effects of SGLT2 inhibitors may be augmented by plant-based diets to improve glomerular hemodynamics and restore autophagy [194].

KDGO guidelines recommend a low-protein diet and plant-based regimens to delay end-stage renal disease and to improve the quality of life. Nutritional interventions including lower dietary protein and sodium have been recommended for those with one functional kidney, such as kidney donors and children born with solitary kidneys [313]. Diets with moderately low protein supplemented with keto acids and amino acids have been formulated for pregnant women with CKD and FSGS [314]. Plant food-based dietary formulations may lower hyperfiltration to prolong kidney function in diabetes, hypertension, and obesity [315,316,317,318]. Systematic trials using low-protein diets with RAAS inhibitors will be valuable for determining their additive effects on albuminuria and glomerulosclerosis [319].

#### 10.6.2. Dietary ω-3 PUFA

High dietary intake of ω-6 PUFA (as linoleic acid, LA), i.e., 80–90% of total PUFA intake, alters the balance between ω-6 and ω-3 PUFA, distorts the balance of LA to α-linolenic acid, EPA and DHA and contributes to the high incidence of chronic diseases. Excessive LA in blood and tissues blocks the availability of ω-3 PUFA for cellular structure and functions [320]. Therefore, ω-3 PUFA supplements are used as adjunctive therapy to treat CKD. A meta-analysis of nine randomized trials showed that ω-3 PUFA supplementation lowered the incidence of end-stage renal disease and slowed disease progression [321]. Another meta-analysis of published articles reported improvement in cardiometabolic parameters in CKD patients [322]. Growing research on ω-3 PUFA will likely lead to products for other specific applications [323].

### 10.7. Flavonoids

Flavonoids are plant-derived compounds with anti-inflammatory, antihypertensive and antidiabetic properties. Many flavonoids are renoprotective in glomerulonephritis, diabetic nephropathy and chemically induced kidney insufficiency. Flavonoids lower blood pressure through diuresis and natriuresis and directly interact with renal parenchyma. Flavonoids act in the reorganization of the renal endothelial barrier, lowering of urinary microalbumin and glomerular hyperfiltration. Flavonoids also attenuate the effects of high fructose consumption, high dietary fat and diabetes [324].

### 10.8. Novel Compounds to Target the Endocannabinoid System (ECS)

Compounds that target the central ECS cause adverse psychological effects. Therefore, new compounds targeting the peripheral ECS are in pre-clinical studies to alleviate the pathophysiology of obesity [325,326]. A combination of peripheral CB1R antagonist and CB2R agonist [327], and several agonists of CB2R [259,328] have shown protection against deterioration of renal function and are considered potential new treatments of diabetic kidney disease.

### 10.9. Other Drugs and Novel Biologicals

Kidney disease of diverse etiologies converge at fibrosis leading to ESRD. Fibrosis links several signaling pathways, including TGF-β, MAPK, Wnt/b-catenin, PI3K/Akt, JAK/STAT and Notch. New drugs, including Pirfenidone and Finerenone are potential new treatments for fibrosis [329]. The peptide hormone relaxin is also emerging as a potent antifibrotic therapy [330]. PPARγ upregulation has also been shown to act as an anti-fibrotic [307,308,331]. Lipoxins, resolvins, protectins and maresins are specialized pro-resolving mediators that stimulate regulated innate responses to resolve inflammation and protect organs. Resolvins have shown potential application in treating chronic kidney disease [332,333,334].

## 11. Summary, Conclusions and Future Directions

Glomerular hyperfiltration is an early event in several diseases leading to CKD and has been studied mainly in diabetes (pre-diabetes) or hypertension and obesity. Hyperfiltration initially causes glomerular stress especially in podocytes but the significance of tubular changes is increasingly recognized. Chronic hyperfiltration leads to irreversible loss of structure and function.

We and several other research groups are addressing biomechanical forces as the drivers of hyperfiltration-induced renal changes. Biomechanical stress disturbs the membrane homeostasis causing the release of PUFA from membrane lipids. PUFA serve as the primary source of early lipid mediators of mechanotransduction and inflammatory response. PUFA from both ω-6 and ω-3 families compete for the same COX, LOX and CYP450 enzymes to generate mediators that trigger cellular signaling. Omega-6 PUFA arachidonic acid generates anti-inflammatory lipoxins in addition to the known pro-inflammatory prostaglandins, thromboxane and leukotrienes. We have demonstrated the significance of the COX2-PGE_2_-EP_2_ signaling axis in mechanotransduction of early cellular stress caused by hyperfiltration-associated shear stress.

Research on the significance of ω-3 PUFA and their metabolites in hyperfiltration is relatively recent. Available information shows the renoprotective effects of ω-3 PUFA and their metabolites. Additionally, ω-3 PUFA also generate specialized pro-resolving molecules as the onsite agents for resolving inflammation. Both ω-6 and ω-3 PUFA are constituents of endocannabinoids that function as pro- and anti-inflammatory agents.

Novel treatments and adjunct therapies may modify the course of the disease due to their effect on hyperfiltration. While many studies refer to hyperfiltration as an early event, its potential as an early indicator of kidney disease has not been fully explored. In this regard, specific lipids may be useful as early biomarkers. Emerging data from long-term follow up of kidney donors and children born with single kidneys will likely elaborate the role of biomechanical forces in hyperfiltration and provide information to characterize biomarkers of hyperfiltration.

## Figures and Tables

**Table 1:** Hyperfiltration is an early event in several kidney diseases.

Pathophysiology Associated with Hyperfiltration/Kidney Disease	References
High dietary protein consumption by vulnerable groups	[18,19,20]
Obesity	[21,22,23,24,25,26,27,28,29]
Diabetes	[30,31,32,33,34,35,36,37,38,39,40,41,42,43,44]
Hypertension	[45,46,47,48,49,50,51,52]
Primary hyperaldosteronism	[53,54,55,56]
Non-alcoholic fatty liver disease (NAFLD)	[57,58,59,60,61,62]
CKD in Kidney donors	[14,17,63,64,65,66,67,68]
CKD in Children born with single functioning kidney or low number of functional nephrons due to other Congenital Anomalies of the Kidney and Urinary Tract (CAKUT)	[16,69,70,71,72,73]
Autosomal Dominant Polycystic Kidney Disease (ADPKD)	[74]
Secondary focal segmental glomerulosclerosis (FSGS)	[75,76,77,78]
Sickle cell Disease (SCD) and glomerular sclerosis	[79,80,81,82]
Cyanotic congenital heart disease/critical congenital heart disease (CCHD)	[83,84,85,86,87]
‘Autoimmune activation’ and inflammation	[88,89]
High altitude renal syndrome	[90]
Dementia	[91]
Stroke	[92,93]
